# Assessment of Social Functioning in Patients With Schizophrenia and Their First-Degree Relatives

**DOI:** 10.14740/jocmr6431

**Published:** 2026-01-04

**Authors:** Takamitsu Shimada, Hiroaki Kihara, Yusuke Nitta, Tatsuya Nagasawa, Mitsuru Hasegawa, Yoshiki Maeda, Yasuhiro Kawasaki, Takashi Uehara

**Affiliations:** a Department of Neuropsychiatry, Kanazawa Medical University, Uchinada, Kahoku-gun, Ishikawa 920-0293, Japan; b Medical Corporation Sekijinkai Okabe Hospital, Kanazawa, Ishikawa 921-8114, Japan

**Keywords:** Schizophrenia, First-degree relatives, Social functioning, Japanese version of Social Functioning Scale, Genetic involvement

## Abstract

**Background:**

Impaired social functioning is one of the core symptoms of schizophrenia (SCZ). Genetic factors have also been implicated in SCZ. To contribute to the discussion on the involvement of genetic factors in SCZ, we evaluated the social functioning of first-degree relatives (FR) of patients with SCZ.

**Methods:**

This was a non-interventional observational study. We examined social functioning using the Japanese version of the Social Functioning Scale (SFS-J) in three groups: SCZ, SCZ FR, and healthy controls (HC). The effects of the groups (SCZ, FR, and HC) on social functioning were evaluated using analysis of covariance. In addition, the cutoff value for SCZ in the SFS total score was calculated, and the trend in the proportion of individuals below the cutoff value in each group was evaluated.

**Results:**

Data from 256 subjects (SCZ (n = 44), FR (n = 26), and HC (n = 186)) were analyzed. Group, years of education, intelligence quotient (IQ), and sex were found to be significant factors affecting SFS total scores. The proportion of SFS scores < 140 (the cutoff value for SCZ) was 9.1% in HC, 57.7% in FR, and 95.4% in SCZ, showing a continuous increase in the proportion of SFS scores < 140 across the three groups (P < 0.0001).

**Conclusions:**

In social functioning assessed by SFS, the score for FR was intermediate between those of SCZ and HC. The results of this study suggest that genetic factors may influence social functioning scores in SCZ and FR.

## Introduction

Schizophrenia (SCZ) is a chronic, relapsing mental disorder characterized by positive symptoms, such as hallucinations and delusions; negative symptoms, such as loss of motivation and emotional expression; and cognitive impairment [1]. The treatment outcome is not always satisfactory, with Jaaskelainen et al reporting that only 13.5% of patients achieved the clinical and social recovery criteria [2]. According to a domestic survey, 70.8% of patients were unmarried, 48.9% were unemployed, and the quality of life (QOL) was particularly low when they also had sleep disorders, depression, or anxiety disorders [3]. SCZ most often develops in late adolescence or early adulthood [4], and its lifetime prevalence is reported to be 0.87% overseas and 0.59% in Japan [3, 5].

Functional outcomes refer to the ability to cope with everyday life events, such as living independently, managing finances, employment, and social activities [6, 7]; these are often impaired in SCZ. SCZ also involves impairments in social functioning, defined as an individual’s ability to adequately perform everyday social tasks and maintain an appropriate social life [8]. Social dysfunction is defined as the inability to adapt flexibly to various social situations and demands [3]. Impaired social functioning is a core symptom of SCZ [9]. Impaired social functions exist regardless of the illness course (first episode or chronic) of SCZ [10, 11]. Individuals at a clinically high risk for psychosis show impairments in social functions even during the prepsychotic phase of SCZ, suggesting that social deficits are present long before the onset of psychotic symptoms [12]. Premorbid impairments in social function showed familial aggregation, implying the possible heritability of social function [13].

Genetic factors are involved in the development of SCZ, with an estimated heritability of 64-81% [14, 15]. Studies of families and twins of patients with SCZ have shown that the more genes shared, such as between first-degree parents or identical twins, the higher the risk of developing SCZ [16, 17]. In particular, the involvement of single nucleotide polymorphisms (SNPs) and copy number variants has been a focus of research; Ripke et al. reported that more than 8,000 SNPs may be involved in SCZ [18]. Although no SNPs have been proven to cause SCZ, it has been suggested that SNPs in *ZNF804A* may affect cognitive function depending on the patient’s intelligence quotient (IQ) [19, 20]. In a study of patients with SCZ and their relatives, Los et al found that the neurocognitive function of unaffected siblings was intermediate between those of patients with SCZ and healthy controls (HC) [21]. In addition, Lin et al reported that the first-degree relatives (FR) of patients with SCZ who did not develop SCZ had significantly lower prospective memory than that of HC [22]. Furthermore, highly reproducible endophenotypes in SCZ, such as P50, P300, and mismatch negativity, which are electrophysiological indices in response to specific brain stimuli, have been reported [23, 24]. Nevertheless, Kerr-Gaffney et al reported that FR of SCZ patients did not differ significantly from HC in terms of impulsivity, a concept that includes both cognitive and behavioral components [25], suggesting that there may not be clear differences between SCZ and its FRs depending on the indicators. To date, there has been insufficient research into the social functioning of patients with SCZ and their relatives. Against this background, we investigated the social functioning of patients with SCZ, their FR, and HC.

## Materials and Methods

### Study design and subject

This was a non-interventional observational study. Patients with SCZ and FR were recruited from both the outpatient and inpatient populations at Kanazawa Medical University Hospital and Okabe Hospital. Patients with SCZ were diagnosed by at least two trained psychiatrists based on unstructured clinical interviews, medical records, and clinical conferences. Patients were diagnosed according to the criteria of the Diagnostic and Statistical Manual of Mental Disorders, Fifth Edition (DSM-5). Unaffected FR were evaluated using the non-patient version of the Structured Clinical Interview for DSM-IV (SCID) to exclude individuals with a current or history of receiving psychiatric services or psychiatric medications. HC were recruited through local advertisements and from staff at Okabe hospitals; they were also evaluated using the non-patient version of the SCID to exclude individuals who had current or past contact with psychiatric services, had received psychiatric medications, or had a family history of any neuropsychiatric diseases within second-degree relatives. Participants were excluded if they had neurological or medical conditions that could affect the central nervous system, including atypical headache, head trauma with loss of consciousness, thyroid disease, chronic hepatic disease, chronic lung disease, kidney disease, active cancer, cerebrovascular disease, epilepsy, seizures, substance-related disorders, or intellectual disorders.

Written informed consent was obtained from all the participants after the procedures were fully explained. This study was performed in accordance with the Declaration of Helsinki of the World Medical Association and approved by the Research Ethics Committee of Kanazawa Medical University (approval number: 1716).

### Endpoints

In this study, social functioning was assessed using the Social Functioning Scale (SFS) [26]. This study examined: 1) the impact of differences between groups (SCZ, FR, and HC) on social functioning; 2) trends in social functioning among each group; 3) a comparison of the SFS subscales among each group; and 4) correlation of SFS scores between patients with SCZ and their unaffected parents.

### Assessment of premorbid IQ and social functioning

Premorbid IQ was assessed for each of the SCZ, FR, and HC groups using the Japanese Adult Reading Test 50 (JART50) [27]. Social function was assessed using the Japanese version of the Social Functioning Scale (SFS-J), which is a self-report questionnaire [28]. The SFS has seven subscales: 1) withdrawal (time spent alone, initiation of conversation, and social avoidance) with score range of 0–15; 2) interpersonal communication (number of friends/having a romantic partner and quality of communication) with score range of 0–12; 3) independence-performance (performance of skills necessary for independent living) with score range of 0–39; 4) independence-competence (ability to perform skills necessary for independent living) with score range of 0–39; 5) recreation (engagement in a range of common hobbies, interests, pastimes, etc.) with score range of 0–45; 6) prosocial activities (engagement in a range of common social activities, e.g., sport) with score range of 0–66; and 7) employment/occupation (engagement in productive employment or a structured program with daily activities) with score range of 0–10. The total score is the sum of the seven domain scores, with a score range of 0–226 [26]. A higher score indicates a higher level of social functioning.

### Statistics

Descriptive statistics were expressed as n (%), mean ± standard deviation (SD). The effect of group on social functioning was evaluated using analysis of covariance (ANCOVA), with the SFS total score as the dependent variable and sex, group, age, years of education, and JART50 as independent variables. The participants were divided into two groups: those with SCZ and others (FR and HC). The cutoff value for SCZ in the SFS total score was calculated using receiver operating characteristic (ROC) analysis. The proportion of participants in each group whose SFS total score was below the cutoff value was evaluated for trends among the three groups using the Cochran-Armitage test. Multiple comparisons were performed among the three groups using the Kruskal-Wallis test for each score on the SFS subscale, and pairwise Wilcoxon tests were performed if significant differences were found. The association between SFS scores of patients with SCZ and their unaffected parents was assessed using the Spearman rank correlation coefficient. The significance level was set at 5%. Statistical analyses were performed using JMP version 18.0.1 (SAS Institute Inc., Cary, NC, USA).

## Results

### Subject background

Data from 256 subjects were analyzed. Among all, 37.5% of the subjects were male, with age of 48.0 ± 16.0 years, educational history of 13.5 ± 2.2 years, JART50-estimated IQ of 106.3 ± 9.2, and a SFS total score of 149.2 ± 36.2 (Table 1). There were significant differences among the three groups in terms of age, educational background, JART50 score, and SFS total score.

**Table 1:** Subjects Background

	Total (n = 256)	SCZ (n = 44)	FR (n = 26)	HC (n = 186)	P
Sex (male), n (%)	96 (37.5%)	17 (38.6%)	6 (23.1%)	73 (39.2%)	0.2761
Age (years), mean ± SD	48.0 ± 16.0	52.7 ± 13.7	68.3 ± 11.1	44.0 ± 14.6	< 0.0001
Educational history (years), mean ± SD	13.5 ± 2.2	12.3 ± 2.0	12.4 ± 1.8	13.9 ± 2.1	< 0.0001
JART50, mean ± SD	106.3 ± 9.2	98.3 ± 11.5	105.0 ± 10.3	108.3 ± 7.2	< 0.0001
SFS total score, mean ± SD	149.2 ± 36.2	90.9 ± 36.5	130.1 ± 24.6	165.7 ± 16.8	< 0.0001

SCZ: schizophrenia; FR: first-degree relatives; HC: healthy controls; JART: Japan Adult Reading Test, SFS: Social Functioning Scale.

### The impact of groups (SCZ, FR, and HC) on social functioning

Significant factors affecting the SFS total score were group, years of education, JART50 predicted IQ, and sex (Table 2). Factors that increased the SFS total score were HC, years of education, JART50 predicted IQ, and female sex, whereas factors that decreased it were FR, SCZ, and male sex. The difference (± standard error) in SFS total score was –27.3 ± 5.7 (P = 0.0067) between SCZ and FR, –30.9 ± 4.9 (P = 0.0004) between FR and HC, and –58.2 ± 4.1 (P < 0.0001) between SCZ and HC (Table 3).

**Table 2:** Effect of Group (SCZ, FR, HC) on SFS Total Score: ANCOVA

	Estimate	SE	*t* value	P
Group (HC)	29.71	2.32	12.8	< 0.0001
Group (FR)	–1.21	3.25	–0.37	0.711
Group (SCZ)	–28.5	2.88	–9.9	< 0.0001
Age	–0.12	0.09	–1.25	0.2113
Educational history	1.95	0.66	2.97	0.0033
JART50	0.40	0.18	2.23	0.0269
Sex (male)	–3.38	1.34	–2.52	0.0123
Sex (female)	3.38	1.34	2.52	0.0123

Model: P < 0.0001, R^2^ = 0.700. ANCOVA: analysis of covariance; HC: healthy controls; FR: first-degree relatives; SCZ: schizophrenia; SE: standard error; JART: Japan Adult Reading Test.

**Table 3:** Difference in SFS Total Score Between Groups: Linear Contrast Test Based on ANCOVA

	Difference in SFS total score ± SE	P
Difference between SCZ and FR	–27.3 ± 5.7	0.0067
Difference between FR and HC	–30.9 ± 4.9	0.0004
Difference between SCZ and HC	–58.2 ± 4.1	< 0.0001

ANCOVA: analysis of covariance; SCZ: schizophrenia; FR: first-degree relatives; HC: healthy controls.

### Intergroup trends in social functioning

**Figure 1:**
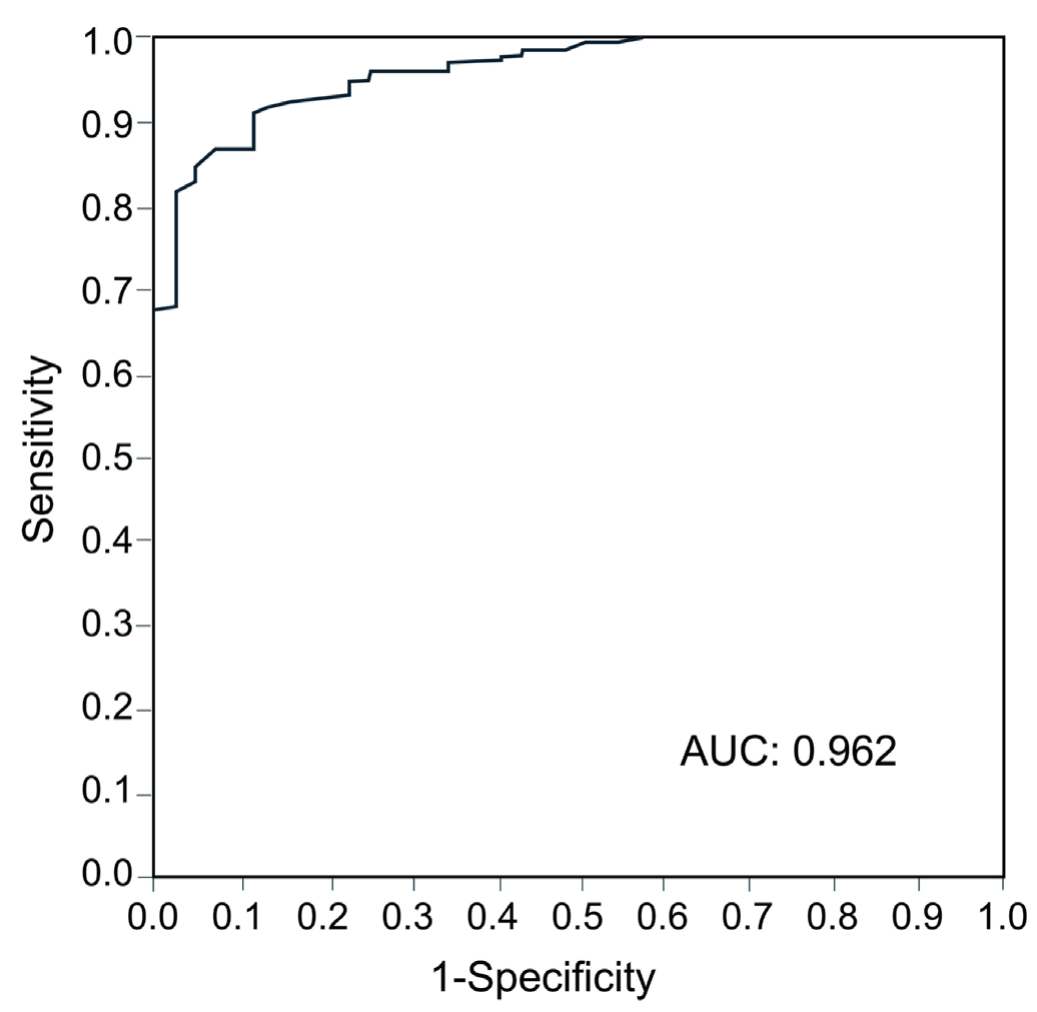
Area under the receiver operating characteristic curve for calculating the cutoff value of SFS for schizophrenia. AUC: area under the curve; SFS: Social Functioning Scale.

**Figure 2:**
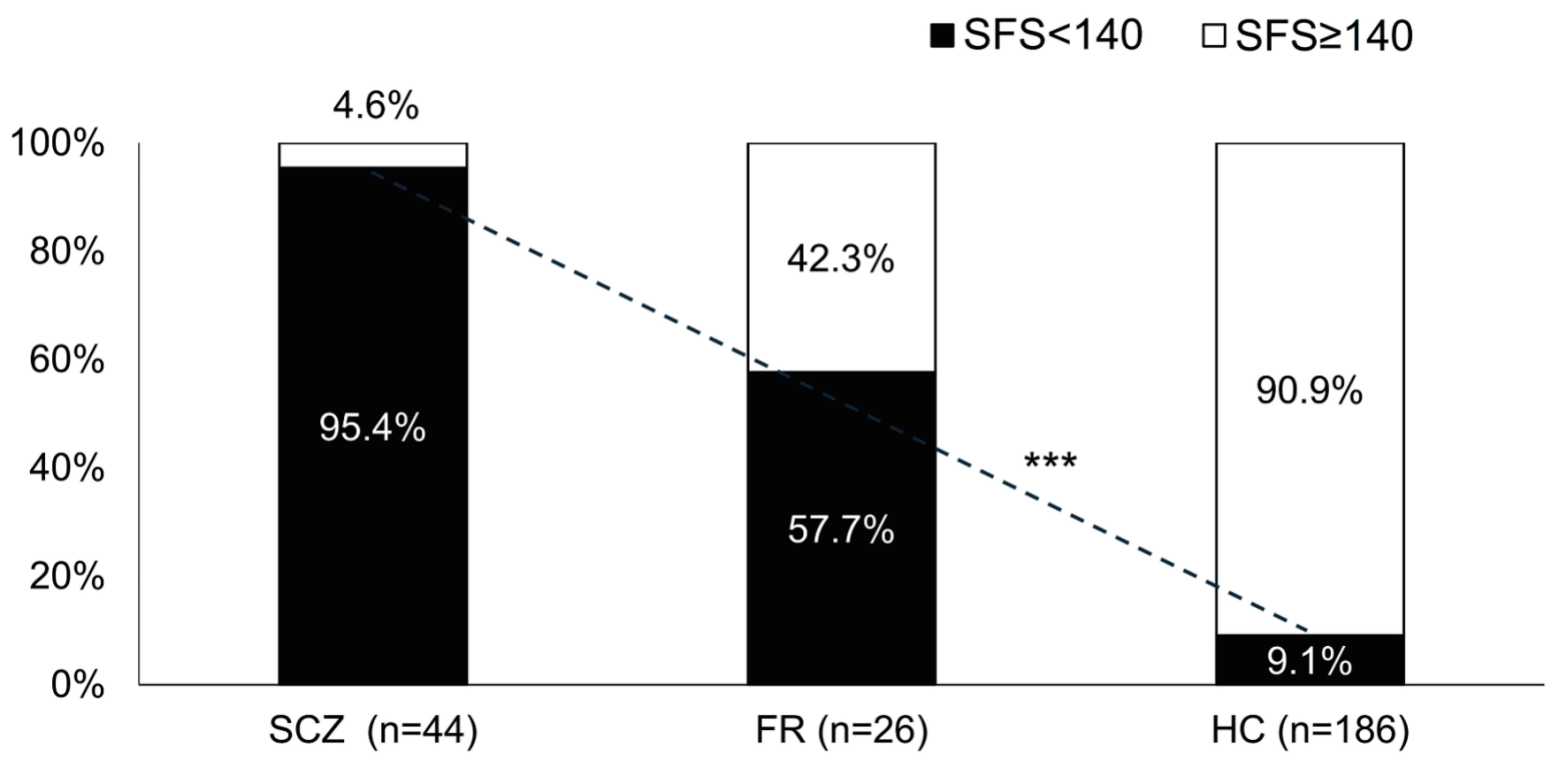
Proportion of subjects with SFS < 140 in each group. ***P < 0.0001 Cochran-Armitage test. There was a significant linear trend in the proportion of SFS < 140 among the three groups. SCZ: schizophrenia; FR: first-degree relatives; HC: healthy controls; SFS: Social Functioning Scale.

ROC curve analysis of the SFS total score for SCZ was performed (AUC 0.962) (Fig. 1). The cutoff value of the SFS total score for SCZ was 140, with a sensitivity of 0.954 and a specificity of 0.849 (Table 4). The proportion of subjects with a SFS total score < 140 was 9.1% in HC, 57.7% in FR, and 95.4% in SCZ, and there was a significant trend for the proportion of patients with SFS < 140 to increase continuously among the three groups (P < 0.0001) (Fig. 2).

**Table 4:** Cutoff Value for SCZ in the SFS Total Score

	SCZ (n = 44)	Other than SCZ (n = 212)	Sensitivity	Specificity
SFS < 140	42 (95.5%)	32 (15.1%)	0.954	0.849
SFS ≥ 140	2 (4.5%)	180 (84.9%)		

SCZ: schizophrenia; Other than SCZ: first-degree relatives and healthy controls; SFS: Social Functioning Scale total score.

### SFS subscale by group

**Figure 3:**
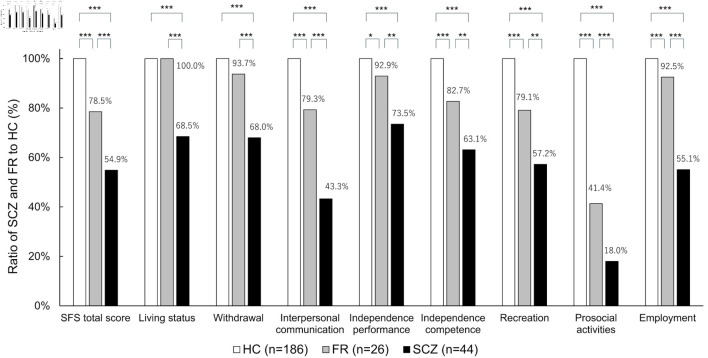
SFS subscales for each group. *P < 0.05, **P < 0.01, ***P < 0.001. HC: healthy controls; FR: first-degree relatives; SCZ: schizophrenia; SFS: Social Functioning Scale.

Figure 3 shows the scores of the SFS subscales for FR and SCZ, using HC as the reference. No significant differences were found between HC and FR in the living status and withdrawal; however, for all other items, scores of FR were significantly lower than those of HC, and scores of SCZ were significantly lower than those of FR.

### The correlation between SFS scores of patients with SCZ and their unaffected parents

**Figure 4:**
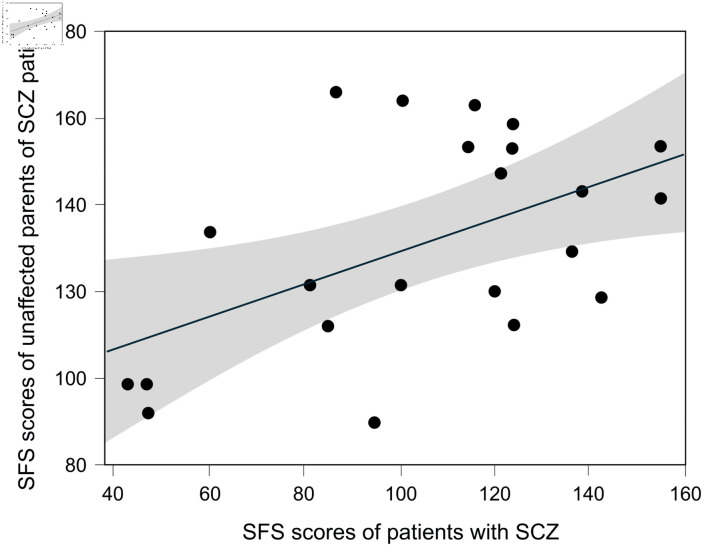
The correlation between SFS scores of SCZ patients and their unaffected parents. A total of 22 pairs comprising patients with SCZ and their unaffected parents were included. The Spearman rank correlation coefficient was 0.3963 (P = 0.0679). The gray shaded area represents the 95% confidence interval. SCZ: schizophrenia.

There were 22 pairs with SFS data for both the patients with SCZ and their unaffected parent. The results showed a tendency for correlation between the SFS scores of patients with SCZ and their unaffected parents, but it was not statistically significant (Spearman rank correlation coefficient: 0.3963, P = 0.0679) (Fig. 4).

## Discussion

In this study, social functioning assessed by the SFS was found to be lower in FR than in HC and higher in FR than in SCZ. In addition, for all SFS sub-items, except for living status and withdrawal, the FR scores were intermediate between those of HC and SCZ. These results indicate that FR differs from HC across a broad range of social functioning domains. As mentioned above, no genetic mutations or SNPs have been identified as the etiology of SCZ; however, many genetic mutations and SNPs have been suggested to be involved [18]. The results of this study indicate that FR, who share genes with patients with SCZ, exhibit characteristics similar to those of SCZ, suggesting that various genetic factors may be involved in the pathogenesis of SCZ. Regarding FR of SCZ, it has been reported that social cognition, working memory [29], prospective memory [22], neurocognitive function [30], and executive function [31] are impaired; this study showed that social function is also impaired. To the best of our knowledge, this study is the first to examine social functioning in SCZ, FR, and HC.

On the other hand, children’s social functioning is influenced by environment during their development. It has been reported that social skills and sociability in parents and children are linked [32, 33]. Social functioning in SCZ patients can be improved through training [34], and children’s social development is influenced by parental nurturing behaviors [35]. Therefore, the SFS as an indicator of social functioning may be influenced not only by genetic factors but also by environmental factors. In this study, we evaluated the correlation of SFS scores in 22 pairs consisting of patients with SCZ and their unaffected parents. The results showed a tendency for correlation between parent and child SFS scores, but the correlation was not significant (Spearman rank correlation coefficient: 0.3963, P = 0.0679). This suggests that, although parent-child environmental factors are involved in SFS in this study, SFS cannot be explained solely by environmental factors. Therefore, regarding the results of this study, while the influence of environmental factors on SFS cannot be denied, this does not negate the possibility of genetic factors being involved.

Among the SFS subscales, the greatest discrepancy in the HC scores was observed in the prosocial activity domain. The proportion of prosocial activity scores relative to the HC was 18.0% for the SCZ group and 41.4% for the FR group. Saris et al reported that the subscale of the SFS that showed the greatest discrepancy between HC and patients with SCZ was prosocial activity [9], which is consistent with the results of our study. Prosocial activities were assessed based on the participant’s activities over the past 3 months, including going out, attending movies, theater performances, sports events, and social gatherings. Patients with SCZ are reported to engage less frequently in deep personal conversations in daily life and are more prone to interpersonal conflict [36]. Considering these factors, the decline in social functioning observed in patients with SCZ or FR may be influenced by impairments in communication skills and ability to build interpersonal relationships.

JART50 (IQ) was an influential factor on the SFS total score. Ohi et al reported that, in patients with SCZ, IQ assessed using the Short Form of the Wechsler Adult Intelligence Scale (WAIS-III SF) was significantly correlated with social activity assessed using the Social Activity Assessment [37]. Leeson et al also reported that IQ was a significant predictor of social functioning in patients with SCZ [38]. Improvement in IQ during the course of SCZ has been suggested to be associated with a better prognosis, and IQ may play a role in various pathophysiological aspects of SCZ [39]. The results of the present study are consistent with those of previous studies.

In our study, the length of education was associated with the SFS total score. SCZ is associated with genetic and environmental factors, and Luo et al reported that increased years of education reduce the risk of SCZ [40]. The results of the present study are consistent with those reported by Luo et al. Although this study could not fully elucidate the causal relationship between educational attainment and social functioning, previous reports have suggested that education may enhance attention, executive function [41], and cognitive function [42]. Therefore, it is possible that the indirect effects of education contributed to improvements in social functioning.

Male sex was a factor that reduced the SFS total score. In our study, the mean total length of hospitalization for patients with SCZ was 275 months for males and 176 months for females (data not shown). The decline in social functioning in men may be due to the fact that more severe cases were included among male patients.

This study has some limitations. First, social functioning in patients with SCZ is thought to be influenced by factors such as cognitive function [1], positive and negative symptoms [43, 44], and treatment-free periods [45]. However, these data were not collected for FR and HC in this study, and therefore these factors were not taken into account when assessing the impact of group on social functioning. Second, we could not exclude the possibility of some methodological selection bias in our samples. Since 88.5% of FR consisted of the parents of SCZ, the mean age in FR was increased. Third, compared with the sample size of HC, those of SCZ and FR were relatively small. Thus, false positive findings are possible. Future studies attempting to replicate our findings using larger SCZ and FR sample sizes are needed. And finally, genetic testing was not performed in this study. Elucidating the genes related to social functioning in SCZ remains a challenge for the future. These limitations should be considered when interpreting the findings.

### Conclusions

In terms of social functioning as assessed by the SFS, scores for individuals with FR were intermediate between those of patients with SCZ and HC. The findings of this study indicate that there may be a potential genetic contribution to SCZ as reflected in social functioning.

## Data Availability

Any queries regarding the availability of data supporting this study should be directed to the corresponding author.

## Abbreviations

ANCOVA: analysis of covariance
AUC: area under the curve
DSM-5: Diagnostic and Statistical Manual of Mental Disorders, Fifth Edition
FR: first-degree relatives
HC: healthy controls
IQ: intelligence quotient
JART 50: Japanese Adult Reading Test 50
QOL: quality of life
ROC: receiver operating characteristic
SCID: Structured Clinical Interview for DSM-IV
SCZ: schizophrenia
SFS-J: Japanese version of the Social Functioning Scale
SNPs: single nucleotide polymorphisms
